# Short-Term Risk of Complications Related to Obstructive Sleep Apnea After Sinonasal Surgery

**DOI:** 10.31486/toj.25.0013

**Published:** 2025

**Authors:** Nrusheel Kattar, Dylan A. Levy, Edward D. McCoul

**Affiliations:** ^1^ Department of Otolaryngology-Head and Neck Surgery, LSU Health Shreveport, Shreveport, LA; ^2^ Department of Otolaryngology-Head and Neck Surgery, Tulane University, New Orleans, LA; ^3^ Department of Otorhinolaryngology and Communication Sciences, Ochsner Clinic Foundation, New Orleans, LA; ^4^ The University of Queensland Medical School, Ochsner Clinical School, New Orleans, LA

**Keywords:** *Intraoperative complications*, *nasal surgical procedures*, *postoperative complications*, *respiratory insufficiency*, *sleep apnea–obstructive*, *surgical procedures–operative*

## Abstract

**Background:**

Surgical manipulation in the sinonasal cavity may transiently increase airflow resistance in patients with obstructive sleep apnea (OSA), predisposing them to inspiratory collapse of the oropharynx and/or hypopharynx. We hypothesized that patients with OSA undergoing sinonasal surgery would have higher rates of unplanned postoperative admission and respiratory complications than patients without OSA.

**Methods:**

In this retrospective cohort study, patients ≥18 years undergoing sinonasal surgery were compared to patients having oropharyngeal, laryngeal, otologic, rotator cuff, and inguinal hernia surgery between January 2013 and December 2020. Cohorts were defined by the presence or absence of a preoperative diagnosis of OSA. All outcome variables were measured based on unique patient encounters for surgery; the total number of surgeries for the overall patient population (n=17,373) was greater than the total number of patients (n=11,951).

**Results:**

Study groups consisted of 4,575 sinonasal, 2,301 oropharyngeal, 1,231 laryngeal, 1,353 otologic, 434 rotator cuff, and 2,057 inguinal hernia surgical patients. Among patients undergoing sinonasal surgery, patients with OSA had more unplanned admissions than patients without OSA (risk ratio [RR] 3.12; 95% CI 2.07-4.70). In all surgery groups, postoperative supplemental oxygen therapy was required more often in patients with OSA. Patients with OSA had the greatest incidence of oxygen desaturation after oropharyngeal surgery (RR 1.95; 95% CI 1.34-2.85). Incidence of 30-day readmission did not differ between patients with and without OSA in any surgical group.

**Conclusion:**

In this study, patients with OSA undergoing sinonasal surgery had a greater risk of unplanned hospital admission than patients without OSA, and patients with OSA undergoing oropharyngeal surgery had a greater risk of postoperative desaturation than patients without OSA. Patients with OSA undergoing any surgery had a greater incidence of supplemental oxygen requirement compared to patients without OSA.

## INTRODUCTION

Obstructive sleep apnea (OSA) is an increasingly prevalent condition that is often undiagnosed in patients undergoing surgery.^1^ The prevalence of OSA is estimated to be 10% among 30- to 49-year-old males, 17% among 50- to 70-year-old males, and 9% among 50- to 70-year-old females.^2^ OSA is characterized by dynamic upper airway obstruction during sleep that may lead to perioperative adverse events including oxygen desaturation, pneumonia, difficult reintubation, and longer hospital stays.^3-5^

The reduced cross-sectional area of the sinonasal cavity has been shown to cause increased airflow resistance in patients with OSA, predisposing them to inspiratory collapse of the oropharynx and/or hypopharynx.^6^ Sinonasal surgery, in turn, has been shown to increase upper airway dimensions and improve long-term outcomes in some patients with OSA.^7^ However, in the immediate postoperative period, mucosal edema, sinonasal packing, and bleeding can temporarily reduce the cross-sectional area of the nasal cavity, creating the potential for postoperative respiratory complications in patients with OSA.^8^

A recent (2020) analysis of breathing patterns in patients with OSA used computational fluid dynamics technology to demonstrate that nasal breathing with a closed mouth produces undisturbed and steady airflow, whereas oral breathing leads to a disturbed, unsteady airflow.^9^ This finding suggests that oral breathing is a primary contributor to airway collapse. For patients with OSA and varying degrees of postoperative mucosal edema or increased airflow resistance after sinonasal surgery, the short-term reduction in nasal breathing ability could lead to overdependence on ineffective oral breathing, thereby increasing the risk for airway collapse and potential perioperative complications.

We hypothesized that patients with OSA undergoing sinonasal surgery would have higher rates of unplanned postoperative admission and increased respiratory complications than patients without OSA. We aimed to compare outcomes for patients with OSA after sinonasal surgery to outcomes for patients with OSA after oropharyngeal and laryngeal surgery, procedures that are expected to have a higher incidence of OSA-related adverse events based on literature demonstrating that mucosal sensory impairment in both the oropharynx and larynx contributes to the pathophysiology of OSA.^10^ We also sought to compare these outcomes with otologic and nonotolaryngologic surgeries that are expected to have a lower incidence of OSA-related adverse events than sinonasal surgery.

## METHODS

We conducted a retrospective cohort study of patients ≥18 years of age who underwent outpatient surgery at a tertiary medical center. Participants were selected from the centralized data warehouse maintained by a large regional health system in the southeastern United States. This health system has a heterogenous payor mix that includes patients who receive government insurance and those who are privately insured. Institutional review board approval was obtained from Ochsner Clinic Foundation. STrengthening the Reporting of OBservational studies in Epidemiology (STROBE) criteria were used as a guide for conduct of this study.

The primary group of interest was patients who underwent sinonasal surgery. These subjects were divided according to the presence or absence of a prior diagnosis of OSA. To determine if any observed differences within this primary group of interest were similar among other surgical populations, patients who underwent oropharyngeal, laryngeal, otologic, rotator cuff, or inguinal hernia surgery were also included. Rotator cuff repair and inguinal hernia repair are commonly performed outpatient surgeries that were chosen to contrast with otolaryngologic surgeries because of their lack of manipulation of the upper aerodigestive tract.

Patients in the other surgery groups were similarly subdivided based on a prior diagnosis of OSA. Patients who underwent nonelective (eg, urgent or emergent) procedures and patients with a planned hospital admission were excluded.

### Variables

Independent variables were age, sex, and type of surgery. Outcomes of interest were unplanned postoperative admission, oxygen desaturation (documented pulse oximetry <92%) while in the postanesthesia recovery unit, supplemental oxygen therapy provided during the postoperative period on the day of surgery, and readmission within the first 30 postoperative days. Unplanned postoperative admission was defined as occurring within the same encounter as the surgery, thereby differentiating this variable from the 30-day readmission variable. All the outcome variables were measured based on unique patient encounters for surgery in the electronic health record. Therefore, patients could have more than one encounter if they had multiple surgeries.

### Statistical Methods

Statistical analyses were performed using SPSS Statistics version 25.0 (IBM Corporation). Categorical variables are summarized using frequency and percentage descriptive statistics. Continuous variables are summarized by the median and range if not normally distributed. Normality was assessed using the Shapiro-Wilk test. Categorical variables were compared between groups using Fisher exact test or chi-square test. A paired *t* test was used to compare continuous variables between 2 groups of interest, while a one-way analysis of variance test was used to compare continuous variables between multiple groups. Risk ratio (RR) and the corresponding 95% CI were obtained to compare the likelihood of a primary outcome based on exposure (eg, history of OSA). Comparative analyses between the OSA and non-OSA cohorts were conducted using the Mantel–Haenszel test within a fixed-effects model framework to estimate pooled effect sizes. A *P* value of <0.05 was considered to indicate a significant difference for all statistical tests.

## RESULTS

### Participants

Study groups consisted of 4,575 sinonasal, 2,301 oropharyngeal, 1,231 laryngeal, 1,353 otologic, 434 rotator cuff, and 2,057 inguinal hernia surgical patients. For this retrospective cohort study that used automated data collection from medical records, all patients who fit the inclusion criteria were included.

**Figure 1.**
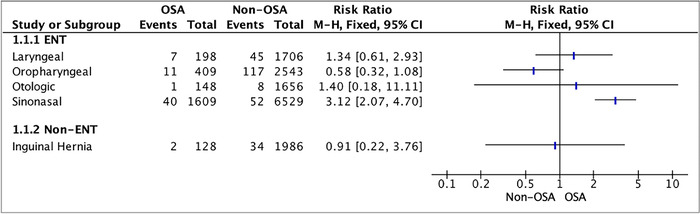
**Forest plot of unplanned hospital admission for patients with and without obstructive sleep apnea by surgery type.** Note: The rotator cuff surgery group is not included in this analysis because none of the patients in the OSA subgroup required unplanned hospital admission and risk comparison was not possible. CI, confidence interval; ENT, ear, nose, and throat; M-H, Mantel-Haenszel; OSA, obstructive sleep apnea.

Table 1 presents baseline characteristics of individual patients, whereas Tables 2 through 5 and Figures 1 through 4 present the outcomes for the unique patient encounters for surgery. As expected, the total number of surgeries (n=17,373) is greater than the total number of patients (n=11,951).

**Table 1.** Baseline Characteristics for Patients With and Without Obstructive Sleep Apnea by Surgery Type, n=11,951

Surgery Type/Patient Characteristics	Patients With Obstructive Sleep Apnea, n=1,486	Patients Without Obstructive Sleep Apnea, n=10,465	*P* Value
Sinonasal, n=4,575	895 (19.6)	3,680 (80.3)	
Age, years, median (range)	49 (18-65)	42 (18-65)	<0.001
Male	563 (62.9)	1,687 (45.8)	<0.001
Oropharyngeal, n=2,301	189 (8.2)	2,112 (91.8)	
Age, years, median (range)	40 (18-65)	29 (18-65)	<0.001
Male	84 (44.4)	815 (38.6)	0.114
Laryngeal, n=1,231	119 (9.7)	1,112 (90.3)	
Age, years, median (range)	55 (18-65)	54 (18-65)	0.235
Male	67 (56.3)	616 (55.4)	0.850
Otologic, n=1,353	104 (7.7)	1,249 (92.3)	
Age, years, median (range)	53 (18-65)	45 (18-65)	<0.001
Male	55 (52.9)	539 (43.2)	0.055
Rotator cuff, n=434	54 (12.4)	380 (87.6)	
Age, years, median (range)	56 (24-65)	55 (18-65)	0.178
Male	32 (59.3)	219 (57.6)	0.492
Inguinal hernia, n=2,057	125 (6.1)	1,932 (93.9)	
Age, years, median (range)	54 (18-65)	55 (18-65)	0.213
Male	104 (83.2)	1,662 (86.0)	0.382

Note: Except for age, data are presented as n (%), with percentages based on the n for the specific type of surgery.

**Table 2.** Unplanned Hospital Admission for Patients With and Without Obstructive Sleep Apnea by Surgery Type

Surgery Type	Patients With Obstructive Sleep Apnea	Patients Without Obstructive Sleep Apnea	*P* Value
Sinonasal	40/1,609 (2.5)	52/6,529 (0.8)	<0.001
Laryngeal	7/198 (3.5)	45/1,706 (2.6)	0.463
Oropharyngeal	11/409 (2.7)	117/2,543 (4.6)	0.078
Otologic	1/148 (0.7)	8/1,656 (0.5)	0.750
Rotator cuff	0/53 (0)	2/408 (0.5)	0.609
Inguinal hernia	2/128 (1.6)	34/1,986 (1.7)	0.899
Test for subgroup differences			0.003

Notes: The total number of surgeries for this patient population (n=17,373) was greater than the total number of patients (n=11,951). For example, the 895 sinonasal surgery patients with OSA underwent 1,609 total surgeries, and the 3,680 sinonasal surgery patients without OSA underwent 6,529 total surgeries. Data are presented as n (%).

### Descriptive Data

Descriptive statistics for patients with and without OSA undergoing the 6 types of surgery are shown in Table 1. Patients with OSA in the sinonasal, oropharyngeal, and otologic surgery groups had a statistically significant greater median age than patients without OSA (*P*<0.001). The sinonasal surgery group had a significantly larger proportion of males in the OSA group compared to the non-OSA group. Among the rotator cuff and inguinal hernia surgery groups, no differences in age or sex were found between the OSA and non-OSA groups.

### Outcome Data

Among patients undergoing sinonasal surgery, those diagnosed with OSA had a greater incidence of unplanned postoperative admission than patients without OSA (RR 3.12; 95% CI 2.07-4.70) (Figure 1). This association was significantly greater than that seen with any other surgery group (Table 2).

**Figure 2.**
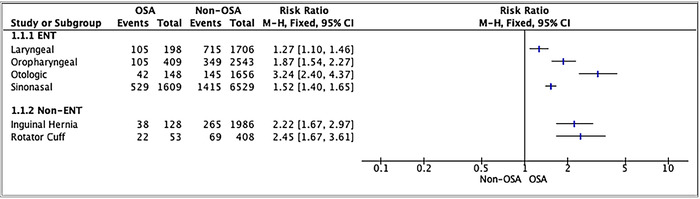
**Forest plot of supplemental oxygen requirement for patients with and without obstructive sleep apnea by surgery type.** CI, confidence interval; ENT, ear, nose, and throat; M-H, Mantel-Haenszel; OSA, obstructive sleep apnea.

A greater proportion of OSA patients required postoperative oxygen supplementation compared to patients without OSA in all surgery groups (Figure 2). Testing for subgroup differences demonstrated that each surgery group varied in magnitude of difference between the OSA and non-OSA cohorts with regard to the supplemental oxygen requirement (Table 3).

**Table 3.** Supplemental Oxygen Requirement for Patients With and Without Obstructive Sleep Apnea by Surgery Type

Surgery Type	Patients With Obstructive Sleep Apnea	Patients Without Obstructive Sleep Apnea	*P* Value
Sinonasal	529/1,609 (32.9)	1,415/6,529 (21.7)	<0.001
Laryngeal	105/198 (53.0)	715/1,706 (41.9)	0.003
Oropharyngeal	105/409 (25.7)	349/2,543 (13.7)	<0.001
Otologic	42/148 (28.4)	145/1,656 (8.8)	<0.001
Rotator cuff	22/53 (41.5)	69/408 (16.9)	<0.001
Inguinal hernia	38/128 (29.7)	265/1,986 (13.3)	<0.001
Test for subgroup differences			<0.001

Notes: The total number of surgeries for this patient population (n=17,373) was greater than the total number of patients (n=11,951). For example, the 895 sinonasal surgery patients with OSA underwent 1,609 total surgeries, and the 3,680 sinonasal surgery patients without OSA underwent 6,529 total surgeries. Data are presented as n (%).

**Figure 3.**
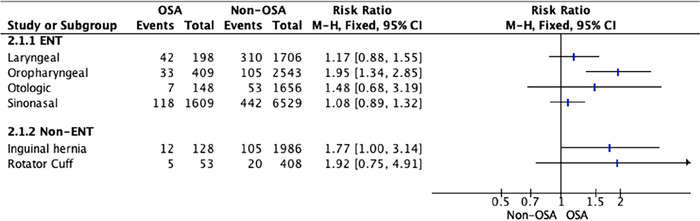
**Forest plot of oxygen desaturation <92% for patients with and without obstructive sleep apnea by surgery type**. CI, confidence interval; ENT, ear, nose, and throat; M-H, Mantel-Haenszel; OSA, obstructive sleep apnea.

Patients with OSA undergoing oropharyngeal surgery had a greater incidence of postoperative oxygen desaturation (<92%) than patients without OSA (RR 1.95; 95% CI 1.34-2.85) (Figure 3). For other types of surgery, postoperative oxygen desaturation was not different between patients with and without OSA (Table 4). Subgroup analysis revealed no statistically significant differences.

**Table 4.** Oxygen Desaturation <92% for Patients With and Without Obstructive Sleep Apnea by Surgery Type

Surgery Type	Patients With Obstructive Sleep Apnea	Patients Without Obstructive Sleep Apnea	*P* Value
Sinonasal	118/1,609 (7.3)	442/6,529 (6.8)	0.423
Laryngeal	42/198 (21.2)	310/1,706 (18.2)	0.297
Oropharyngeal	33/409 (8.1)	105/2,543 (4.1)	<0.001
Otologic	7/148 (4.7)	53/1,656 (3.2)	0.320
Rotator cuff	5/53 (9.4)	20/408 (4.9)	0.171
Inguinal hernia	12/128 (9.4)	105/1,986 (5.3)	0.050
Test for subgroup differences			0.07

Notes: The total number of surgeries for this patient population (n=17,373) was greater than the total number of patients (n=11,951). For example, the 895 sinonasal surgery patients with OSA underwent 1,609 total surgeries, and the 3,680 sinonasal surgery patients without OSA underwent 6,529 total surgeries. Data are presented as n (%).

**Figure 4.**
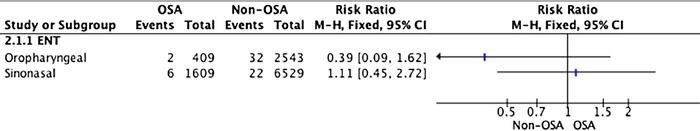
**Forest plot of 30-day readmission for patients with and without obstructive sleep apnea who underwent oropharyngeal and sinonasal surgery.** Note: The laryngeal, otologic, rotator cuff, and inguinal hernia surgery groups are not included in this analysis because none of the patients in the OSA subgroup required readmission within 30 days and risk comparison was not possible. CI, confidence interval; ENT, ear, nose, and throat; M-H, Mantel-Haenszel; OSA, obstructive sleep apnea.

The incidence of readmission did not differ between OSA and non-OSA patients in any surgical group and there was no difference between groups on subgroup analysis (Figure 4, Table 5).

**Table 5.** Thirty-Day Readmission for Patients With and Without Obstructive Sleep Apnea by Surgery Type

Surgery Type	Patients With Obstructive Sleep Apnea	Patients Without Obstructive Sleep Apnea	*P* Value
Sinonasal	6/1,609 (0.4)	22/6,529 (0.3)	0.825
Laryngeal	0/198 (0)	7/1,706 (0.4)	0.367
Oropharyngeal	2/409 (0.5)	32/2,543 (1.3)	0.176
Otologic	0/148 (0)	0/1,656 (0)	–
Rotator cuff	0/53 (0)	1/408 (0.2)	0.718
Inguinal hernia	0/128 (0)	9/1,986 (0.5)	0.445
Test for subgroup differences			0.22

Notes: The total number of surgeries for this patient population (n=17,373) was greater than the total number of patients (n=11,951). For example, the 895 sinonasal surgery patients with OSA underwent 1,609 total surgeries, and the 3,680 sinonasal surgery patients without OSA underwent 6,529 total surgeries. Data are presented as n (%).

## DISCUSSION

Among patients undergoing sinonasal surgery, those with OSA had more unplanned admissions than those without OSA, which is consistent with the hypothesis that sinonasal surgery is associated with a greater incidence of short-term respiratory complications. In all surgery groups, postoperative supplemental oxygen therapy was required more often in patients with OSA. Patients with OSA had the greatest incidence of oxygen desaturation after undergoing oropharyngeal surgery.

The implications of increased unplanned postoperative admission in patients with OSA who undergo sinonasal surgery are significant and may be useful in the perioperative counseling of these patients. Previous evidence has been limited to demonstrating the effectiveness of nasal surgery in reducing daytime sleepiness and snoring in patients with OSA, as well as a possible higher incidence of postoperative hypoxemia in OSA patients undergoing transsphenoidal surgery.^11,12^

This study demonstrated increased supplemental oxygen use in patients with OSA regardless of the type of surgery performed. Previous evidence similarly demonstrated that patients with OSA tend to have increased airway collapse, delayed restoration of airway patency, and more respiratory depression in the postoperative period compared to patients without OSA.^13-15^

Patients with OSA in all the surgical groups in this study, and in the oropharyngeal cohort in particular, had an increased rate of oxygen desaturation <92% compared to patients without OSA. These findings are consistent with the current understanding of the pathophysiology of upper airway surgery. Operative manipulation of the oropharynx can result in postoperative pharyngeal edema that commonly presents after extubation as respiratory compromise requiring oxygen supplementation, especially in patients with previously unrecognized OSA.^16^ Interestingly, we found no significant difference in 30-day readmission rates between the OSA and non-OSA groups for all surgery cohorts, which potentially could be explained physiologically by progressive postoperative resolution of upper airway edema.

Although the treatment of OSA has been studied extensively in pediatric otolaryngology and sleep surgery,^17-19^ this study aimed to address the paucity of evidence on postoperative complications in patients with OSA who undergo otolaryngologic surgery. To our knowledge, this study is the first to examine immediate postoperative outcomes in these patients and comparatively analyze the differences in outcomes between sinonasal, oropharyngeal, laryngeal, otologic, and nonotolaryngologic surgery (rotator cuff repair and inguinal hernia repair).

Current recommendations for perioperative care of OSA patients undergoing surgery include continuous positive airway pressure treatment perioperatively, conservative use of opioids, and nonsupine positioning after surgery.^20-22^ This study adds to the literature by highlighting the potential for adverse perioperative events for patients with OSA who undergo otolaryngologic surgery. These patients can benefit from a preoperative discussion with their otolaryngologist regarding these risks.

### Limitations

Despite the retrospective nature of this project and data collection from medical records review, minimal bias was expected aside from possible systematic bias that may have been introduced during initial chart documentation. The authors believe that the patient population studied is a representative sample of the intended target population and that the variables of interest were readily discernible from patient medical records.

However, this large-scale retrospective study did not permit controlling for age, sex, comorbidities, or severity of OSA between the OSA and non-OSA cohorts in each surgery group. Other factors, such as comorbid heart/lung conditions, baseline respiratory health, body mass index, and smoking status were not uniformly available in the retrospective data to use as controls. Furthermore, because of the retrospective aspect of this study, ascertaining the specific etiology of unplanned admissions or readmissions was difficult. Also, although the majority of the surgeries involved general anesthesia, some of the patients may have received variations such as monitored anesthesia care or regional blocks that could not be accounted for in this design. The possibility also exists that undiagnosed OSA patients were included in the non-OSA group, a situation that could be alleviated with a prospectively designed study comparing polysomnogram-positive and -negative cohorts.

To address limitations in this design, future studies may focus on using datasets of cohorts matched for age, sex, and medical comorbidities with stratification based on objective measures of severity of OSA such as the apnea-hypopnea index.

### Generalizability

Because of the retrospective design, this study avoids the threat to external validity from the Hawthorne effect as no bias is attributable to changing behaviors upon observation.^23^

Although we noted some significant differences in median age between OSA and non-OSA cohorts, these differences are consistent with the epidemiology of OSA demonstrated in the literature.^24,25^ The differences in sample size between the surgery groups could affect generalizability, but a targeted timeframe—such as a 1-year study period—would allow for stratification of other variables such as the apnea-hypopnea index to organize results based on OSA severity.

## CONCLUSION

Patients with OSA undergoing sinonasal and oropharyngeal surgery have a greater risk of unplanned hospital admission and postoperative oxygen desaturation, respectively, than patients without OSA. Patients with OSA undergoing any type of surgery had a greater incidence of supplemental oxygen requirement compared to patients without OSA. Although the implications of these findings for clinical practice require further investigation in the form of prospective matched designs, the results of this study may have utility in otolaryngology with preoperative counseling aimed at increasing the awareness of perioperative adverse events for patients with OSA undergoing sinonasal and oropharyngeal surgery.

## Notes

*This article meets the Accreditation Council for Graduate Medical Education and the American Board of Medical Specialties Maintenance of Certification competencies for Patient Care and Medical Knowledge.*
